# Appraisal of Space Words and Allocation of Emotion Words in Bodily Space

**DOI:** 10.1371/journal.pone.0081688

**Published:** 2013-12-11

**Authors:** Fernando Marmolejo-Ramos, María Rosa Elosúa, Yuki Yamada, Nicholas Francis Hamm, Kimihiro Noguchi

**Affiliations:** 1 School of Psychology, Faculty of Health Sciences, University of Adelaide, Adelaide, Australia; 2 Department of Psychology, National University of Distance Education, Madrid, Spain; 3 Faculty of Arts and Science, Kyushu University, Fukuoka, Japan; 4 Department of Statistics, Colorado State University, Fort Collins, Colorado, United States of America; University of Bologna, Italy

## Abstract

The body-specificity hypothesis (BSH) predicts that right-handers and left-handers allocate positive and negative concepts differently on the horizontal plane, i.e., while left-handers allocate negative concepts on the right-hand side of their bodily space, right-handers allocate such concepts to the left-hand side. Similar research shows that people, in general, tend to allocate positive and negative concepts in upper and lower areas, respectively, in relation to the vertical plane. Further research shows a higher salience of the vertical plane over the horizontal plane in the performance of sensorimotor tasks. The aim of the paper is to examine whether there should be a dominance of the vertical plane over the horizontal plane, not only at a sensorimotor level but also at a conceptual level. In Experiment 1, various participants from diverse linguistic backgrounds were asked to rate the words “up”, “down”, “left”, and “right”. In Experiment 2, right-handed participants from two linguistic backgrounds were asked to allocate emotion words into a square grid divided into four boxes of equal areas. Results suggest that the vertical plane is more salient than the horizontal plane regarding the allocation of emotion words and positively-valenced words were placed in upper locations whereas negatively-valenced words were placed in lower locations. Together, the results lend support to the BSH while also suggesting a higher saliency of the vertical plane over the horizontal plane in the allocation of valenced words.

## Introduction

Various studies from embodied cognition theory suggest that the comprehension of concrete concepts entails the activation of sensorimotor systems (e.g, [1]–[4]). For instance, a phenomenon called the action-sentence compatibility effect (ACE) demonstrates the influence of language on motor actions. Under the ACE paradigm, participants are faster at deploying motor responses to sentences describing the same action than to sentences describing an antagonist action [5]. The influence of motor processes on language comprehension has also been documented. It has been shown that lexical decision responses to words referring to manipulable objects are more accurate when a motor movement is being performed than when no movement is performed [6].

Other evidence indicates that the comprehension of abstract concepts, like emotion words, also calls for the activation for sensorimotor systems [7]–[8]. Wilson and Gibbs [9] showed that performing actual and even imagined body actions, facilitates the comprehension of metaphoric sentences. Ulrich and Maienborn [10] demonstrated that the concepts of “past” and “future” are facilitated when leftward and rightward movements, respectively, are performed. Finally, findings from neurosciences [11]–[12] indicate that the processing of metaphorical sentences activates brain areas related to action planning; however as sentences become more abstract (i.e., literal → metaphoric → abstract), the recruitment of sensorimotor areas tends to diminish [13].

The evidence reviewed thus strongly suggests that sensorimotor systems are likely to be activated during the processing of both concrete and abstract concepts. However, such a claim has been challenged, particularly, from research in neurosciences. For instance, Mahon and Caramazza [12] propose that apraxic subjects cannot perform actions associated with objects, but they are capable of naming them and recognising pantomimes associated with those objects. A radical embodiment theory would predict that impairment in motor processes would affect recognition or the naming of objects, but this is not the case in apraxic subjects in which object recognition and recognition of object-related actions remain unharmed (see also [11]). This sort of evidence indicates that other processes might occur when complete embodiment does not occur. As some recent evidence indicates, it is possible to conceive that sensorimotor representations can be encoded in linguistic forms that serve as a “symbolic bypass” to index embodiment (see [14]–[17]).

This re-appraisal of the embodiment theory has led to the proposal of a graded-embodiment view in which the emphasis is on determining levels of embodiment rather than in determining whether embodiment occurs or not [13], [18]–[20]. It could then be argued that abstract concepts can gain sensorimotor properties via potential levels of association with related concrete concepts. A possible explanation is that abstract concepts can be grounded in concrete concepts via metaphoric mappings (e.g., [21]–[22], [10]).

In relation to the processing of emotion concepts, it has been argued that people rely on spatial perceptions as a mapping metaphor to understand emotion concepts. Metaphors are figures of speech in which an expression is used to refer to something that it does not literally denote in order to suggest a similarity between both. It is notable that, from a linguistic point of view, metaphors not only imply *similarity* between concepts, but also an *association* between them (see [23]). Thus, in linguistic terms, metaphorical processes go hand in hand with metonymical processes, i.e., there are similarities between concepts that enable their selection and there are also associations between concepts that lead to their combination based on past experience.

The study of Casasanto and Dijkstra [24] showed that positively-laden memories were retrieved faster than negatively-laden ones when an upward movement was performed, and the opposite pattern was found when a downward movement was performed. Meier, Moller, Chen, and Riemer-Peltz [21] found that people tend to appraise more positively northerly rather than southerly locations in a city, and that low socio-economic groups are regarded as more likely to reside in southern areas than in northern areas.

Finally, it has been found that positive images that are presented in various locations on a computer screen are recalled as being presented at the top area of the screen, whereas negative images are recalled as being presented at the bottom area of the screen [25]. At the same time, it is useful to clarify that the association between spatial metaphors and emotion concepts seems to be unidirectional in that space is used to represent affect, but not the other way around [26]. The reason for this unidirectionality rests on the fact that abstract concepts, like emotion words, borrow sensorimotor properties from concrete concepts in order to gain understanding. It is difficult then to conceive of how domains with rich sensorimotor properties, like space, would rely on domains which lack them.

Emotion concepts are not only associated with spatial coordinates on the vertical plane; an association between these concepts and the horizontal plane has also been reported. In fact, the body-specificity hypothesis (BSH) predicts that right-handers and left-handers allocate positive and negative concepts differently on the horizontal plane, i.e., while left handers allocate negative concepts on the right-hand side of their bodily space, right-handers allocate such concepts to the left-hand side. Casasanto [27] presents evidence which suggests that people associate valenced concepts with the side of their bodily space on which they are more skilful. In a series of experiments, Casasanto [27] showed that right-handers allocated positive concepts onto their rightward bodily space and negative concepts onto their leftward bodily space, while left-handers exhibited an opposite trend. This association is further supported by neurological studies suggesting an association between the left hemisphere and the processing of positive concepts and the right hemisphere and the processing of negative concepts (see [28]–[29]).

Research in hand laterality tasks complements the findings reported above by suggesting that right-handers are faster to identify right hands than left hands. Left-handers show a reversed pattern but tend to show no facilitation for either hand (see [30]–[31]). Additionally, the evidence indicates that left-handers are less lateralised than right-handers. For instance, performance of participants in the Edinburgh Handedness Inventory has shown that there is a higher degree of lateralisation in right-handers than in left-handers (see [30]). The fact that left-handers are less lateralised than right-handers could be due to the fact that the predominance of right-handers has made left-handers become familiar with right-hand positions or right-hand-prone usages (see [32]).

The literature reviewed suggests that, regardless of handedness, in the vertical plane, the “up” location associates with positive concepts while the “down” spatial location associates with negative concepts. In addition, the findings of Casasanto [27] predict that in the horizontal plane, the “right” spatial location associates with positive concepts and the “left” spatial location associates with negative concepts in the case of right-handers, while in the case of left-handers, this pattern is reversed. These results thus indicate that there seems to be a saliency of the vertical plane over the horizontal plane in that while an association between positiveness and negativeness and locations in the horizontal plane is determined by handedness, such association between valences and locations in the vertical plane is not affected by this factor. Therefore, the saliency of one coordinate plane over the other is understood herein as an association between the coordinate plane and valence. Additionally, such association is not determined by many factors and rather seems to have high generalisability.

There is also evidence suggesting a salience of the vertical plane over the horizontal plane across different sensorimotor modalities such as the haptic, visual, and auditory. For example, it has been reported that tactile exploration of 2D symmetric shapes is facilitated more when they are vertically oriented than when they are horizontally or obliquely oriented (see [33]). Cattaneo, Fantino, Silvanto, Tinti, and Pascual-Leone [34] showed that participants memorise and reproduce symmetric configurations better when they are visually presented along the vertical plane than when they are presented along the horizontal plane. Martin, Flanagan, McAnally, and Eberle [35] have shown that, under specific experimental conditions, sound repetition helps to increase accuracy in the localisation and recall of sounds presented on the vertical auditory plane only, even though sounds presented on these auditory planes seem to be localised and recalled using somehow similar processes. In auditory processing, sounds can be perceived in relation to their elevation (vertical auditory plane, VAP) and azimuth (horizontal auditory plane, HAP); thus, very distinctive experimental factors affect the salience of one plane over the other. Additionally, the cues used to localise sounds in these planes are thought to differ. That is, interaural difference cues are thought to dominate in the horizontal plane and spectral cues are thought to dominate in the vertical plane. For example, it is suggested that sound localisation is better in the horizontal plane than in the vertical plane when target sounds are coupled with background sound [36]. However, since ears are localised on the horizontal plane it is somehow expected that in most cases the localisation of sounds on the HAP will outperform the localisation of sounds on the VAP.

Research on the universality of geographical categories lends extra support to the salience of the vertical plane over the horizontal plane. Mark and Frank [37] argue that “left” and “right” locations are less salient than those of “up” and “down” since people are more likely to confuse East-West than North-South. In addition, Freeman [38] reviews research on the relationship between pictures and sentences proposing that “people refer to the locations of objects positively, where upward and forward from the observer are positive directions” (p.164). Freeman's argument is that a person has a 3D coordinate composed of the natural axes “up”-“down” and “front-“back” that are immediately observable, whereas the “left-“right” axis is less salient since it is equally easy to attend to either direction. This evidence thus favours a higher salience of the vertical plane over the horizontal plane in the case of geographical navigation.

The studies reviewed do not deny the notion of space-emotion association based on handedness, but they indicate a higher salience of the vertical plane over the horizontal plane in relation to sensorimotor processes. However, to our knowledge, the empirical data showing the higher salience of the vertical plane over the horizontal have been obtained in tasks requiring perceptual and motor responses. Thus, complementarily the present study hypothesized that, regardless of handedness, there should be a dominance of the vertical plane over the horizontal plane, not only at a sensorimotor level but also at a conceptual level, using a word allocation task. The appraisal of spatial locations should be more marked on the vertical plane than on the horizontal plane at a sensorimotor level and such appraisal should be reflected in the way spatial concepts are appraised. Furthermore, if there is an association between abstract concepts and sensorimotor systems, it could be claimed that sensorimotor experiences shape the way concepts are appraised in relation to spatial locations. The experiments presented in this research aim to test these claims.

The aim of this work is to determine whether such an association and related saliencies are reflected in the way people appraise the spatial concepts “up”, “down”, “left”, and “right” (Experiment 1), and the way people allocate emotion concepts in spatial locations (Experiment 2). In Experiment 1, we conducted a paper-based study using a large number of participants (n = 2153) from 22 different linguistic backgrounds asking them to rate the words “up”, “down”, “left”, and “right”. In Experiment 2, we employed a computer-based experiment using English and Japanese speakers asking them to allocate emotion words into a square grid divided into four boxes of equal areas. The working hypothesis for Experiment 1 was that right-handers and left-handers would rate the words associated with the vertical plane (i.e., “up” and “down”) more extremely than the words associated with the horizontal plane (i.e., “left” and “right”). In Experiment 1 it was also predicted that whereas both left-handers and right-handers would rate “up” as positive and “down” as negative, the ratings for the words “left” and “right” would be handedness-dependent. That is, right-handers would rate the word “right” as more positive than the word “left”, whereas this pattern would be reversed in the case of left-handers. Another significant aim was to analyse whether left-handers exhibit less horizontal lateralisation than right-handers, as reported in previous research.

## Experiment 1

The goal of the first experiment was to determine whether there are differences in the way the spatial locations “up”, “down”, “left”, and “right” are appraised by a large number of right- and left-handers. More importantly, the experiment aimed at determining whether left- and right-handers assessed locations on the horizontal plane differently and whether there are indications of a salience of the vertical plane over the horizontal plane.

### Ethics statement

The experimental protocol was approved by the University of Adelaide Research Ethics Committee. Following the basic principles of the Declaration of Helsinki, adult participants gave written informed consent. The data used in the experiments reported herein are available upon request to the corresponding author.

### Participants

Two thousand, one-hundred and fifty-three University students and members of the community from 22 different linguistic backgrounds voluntarily participated in the study. The participants answered the questionnaire in their home countries. The criteria adopted to select the 22 groups were a) University students, b) native speakers, and c) speakers of different languages. Table 1 presents the demographics of the participants in the study.

**Table 1:** Demographics of the twenty-two languages tested in the study.

Language	Handedness and Gender				*Total*	Age	
	Right-handed		Left-handed			Range	Mean Age (SD)
	Male	Female	Male	Female			
Arabic	12	25	2	3	*42*	19–47	25.09 (6.47)
Bulgarian	11	34	1	8	*54*	21–60	34.40 (11.32)
Cebuano ^‡^	26	39	1	10	*76*	17–32	19.36 (2.21)
Chinese	61	73	6	2	*142*	18–63	21.92 (4.84)
Dutch	9	46	0	8	*63*	17–36	21.46 (2.91)
English	16	58	4	9	*87*	17–46	22.05 (5.44)
Estonian	8	79	2	5	*94*	19–52	30.93 (8.25)
Finish	66	121	6	11	*204*	18–75	27.50 (9.53)
French	27	107	2	15	*151*	18–48	20.94 (3.12)
German	38	63	6	8	*115*	18–46	23.10 (4.07)
Hebrew	29	7	3	1	*40*	22–62	42.05 (11.77)
Hungarian	31	55	9	3	*98*	18–43	20.29 (3.23)
Ilonggo ^‡^	24	48	2	2	*76*	15–20	18.76 (.66)
Italian	12	61	2	4	*79*	18–38	20.31 (3.31)
Japanese	7	24	6	4	*41*	18–53	20.34 (3.76)
Polish	20	95	2	6	*123*	18–37	19.92 (2.50)
Portuguese	23	106	2	2	*133*	17–48	20.75 (5.16)
Russian	32	113	2	6	*153*	16–26	18.69 (1.75)
Serbian	1	38	1	3	*43*	18–32	19.75 (2.66)
Spanish	50	73	5	11	*139*	18–60	23.24 (4.98)
Tagalog ^‡^	11	57	2	10	*80*	18–35	19.38 (1.92)
Thai	20	90	2	8	*120*	18–28	19.38 (1.42)
***Total***	*534*	*1412*	*68*	*139*	*2153*		
***Total (handedness)***	*Right handers = 1946*		*Left handers = 207*				
***Total (gender)***	*Males = 602*		*Females = 1551*				
***Total age range***						*15–75*	
***Total average age (SD)***							*22.59 (6.94)*

= 2.153). Participants who reported being ambidextrous, bilingual or simply whose answers were illegible were not included in this table. The data correspond to all respondents (N

**Note:** languages signalled with “‡” are Austronesian languages spoken in the Philippines.

### Procedure and Materials

Participants were given a one-page questionnaire in which information about their native language, age, gender, and handedness was sought. To account for handedness, a self-report was used since it has been shown to be a reliable way to determine handedness [39].

Participants were asked to rate the words “up”, “down”, “left”, and “right” on a Likert scale ranging from −4 (very negative) to +4 (very positive) with the following instruction: “Assuming that the following words can have a valence that ranges from ‘very negative’ to ‘very positive’, what valence would you give to each word? Please circle the number you think better represents the word.” The words were centred on the page and presented in the order given above, with the Likert scale provided underneath each word, and the order of presentation of spatial words and the polarities of the Likert scale fixed.

### Design

The design consisted of one dependent and two independent factors. The only dependent variable was that of the ratings for the four different words on the Likert scale. The independent factors were the spatial words (i.e., the rated words), analysed as a within-subjects factor (also called a sub-plot factor), and handedness (left and right). Since the purpose of the study was to find general patterns across linguistic groups, the variable “language” was not factored in.

### Statistical analysis

Recent advances in statistics recommend the use of methods that are improved versions of the classic parametric tests and effect sizes. In this study, a rank-based version of the ANOVA was used along with a nonparametric measure of effect size.

Descriptions of how these novel approaches work can be found in recent references (e.g., [40]–[44]), and a more detailed explanation can be found in Appendix A in File S1 of this paper.

### Results

**Figure 1:**
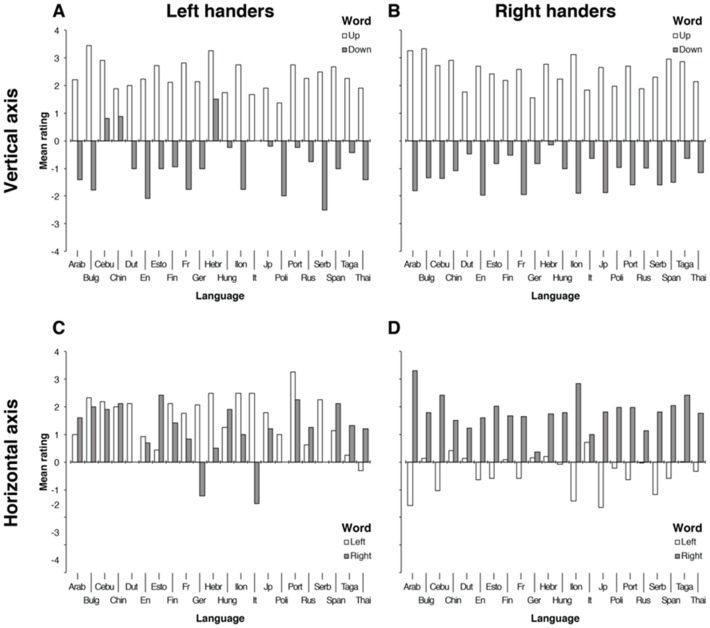
Mean ratings for the spatial words across the twenty-two languages tested. Plots A and B correspond to the mean ratings given by left- and right-handers, respectively, to the words “up” and “down”. Plots C and D correspond to the mean ratings given by left- and right-handers, respectively, to the words “left” and “right”. Languages: Arab  =  Arabic, Bulg  =  Bulgarian, Cebu  =  Cebuano, Chin  =  Chinese, Dut  =  Dutch, En  =  English, Esto  =  Estonian, Fin  =  Finish, Fr  =  French, Ger  =  German, Hebr  =  Hebrew, Hung  =  Hungarian, Ilon  =  Ilonggo, It  =  Italian, Jp  =  Japanese, Poli  =  Polish, Port  =  Portuguese, Rus  =  Russian, Serb  =  Serbian, Span  =  Spanish, Taga  =  Tagalog, Thai  =  Thai.

Figure 1 shows the mean ratings given by left and right-handers in each individual linguistic group for the spatial words.

**Figure 2:**
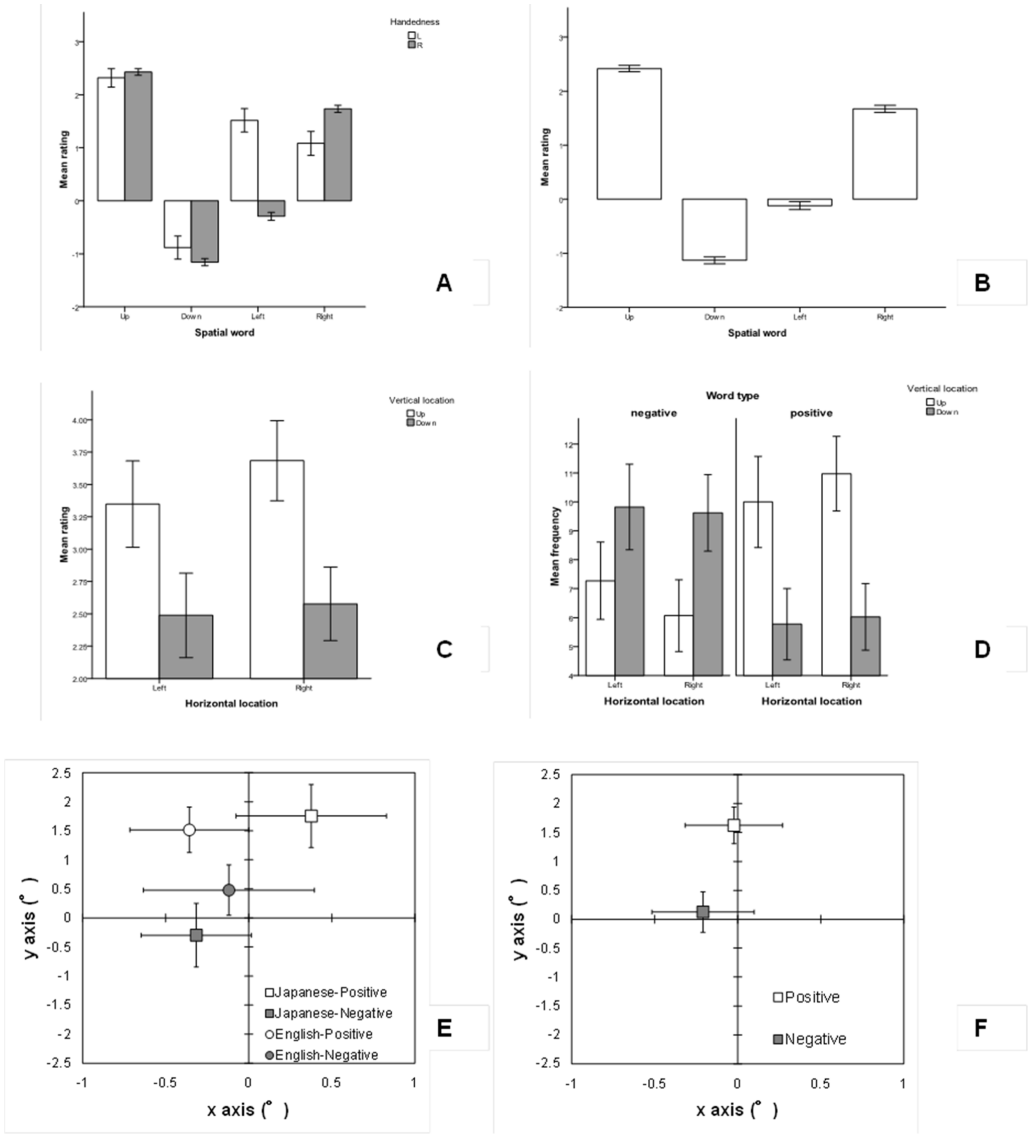
Results of Experiments 1 (A and B) and 2 (C – F). Figures-trait words. Figure C shows the main effect of vertical location in the allocation of valenced words and Figure D shows the average frequency with which words were allocated in the spatial locations given word valence. Figures E and F represent the mean localised positions (X and Y coordinates in visual angles) on the computer screen for negative and positive words according to linguistic group (E) and across languages (F). Error bars represent 95% CIs.

Figures 2A and 2B show the mean ratings given by left and right-handers collated across languages for the spatial words.

Because the ratings are given in a discrete scale, the assumption of continuous data in the commonly used repeated measures ANOVA is violated. In addition, the data indicate that other common assumptions, in particular, normality and homogeneity, also seem to be violated (see Table 2). Thus, we used the ANOVA-type statistic (ATS) for nonparametric repeated measures ANOVA for the analysis of the data (see Appendix A in File S1 for the details). Moreover, two nonparametric dependent and independent sample tests were used in the case of pairwise comparisons. For ATS, we presented its statistic as *F* (*v*_1_, *v*_2_) where *v*_1_ and *v*_2_ are the numerator and denominator degrees of freedom of the *F* distribution. For two-sample tests, we presented the mean (*M*), standard error (*SE*), and its effect size.

**Table 2:** Results of normality and homogeneity tests for the distributions of the ratings of the spatial words.

Spatial word	Normality test		Homogeneity test
	Left handers	Right handers	
Up	*A* = 7.08, *p*<.001	*A* = 74.05, *p*<.001	*L* = .09, *p = *.77
Down	*A* = 3.68, *p*<.001	*A* = 31.19, *p*<.001	*L* = .01, *p = *.91
Left	*A* = 5.66, *p*<.001	*A* = 24.61, *p*<.001	*L* = 1.41, *p = *.24
Right	*A* = 3.37, *p*<.001	*A* = 45.79, *p*<.001	*L* = 6.83, *p<*.01

**Note**. The Anderson-Darling normality test and the robust Brown-Forsythe modification of Levene's test (see Conover, Johnson, & Johnson, 1981) to compare two variances were used. The popular *F*-test (or so-called variance ratio test) is known to be non-robust to small and/or unequal sample sizes and non-normal distributions (Sun, Chernick, & LaBudde, 2011), and hence was not used.

The results showed a main effect of word, *F* (2.63, ∞)  = 507.04, *p*<.001 (*M_up_*  = 2.42, *SE*  = .03; *M_down_*  = −1.13, *SE*  = .04; *M_left_*  = −.12, *SE*  = .04; and *M_right_*  = 1.67, *SE*  = .03), and a main effect of handedness, *F* (1, 236.62)  = 17.49, *p*<.001 (*M_left-handers_*  = 1.01, *SE*  = .07; *M_right-handers_*  = .68, *SE*  = .02 for the mean ratings). There was also a significant interaction between word and handedness, *F* (2.63, ∞)  = 71.88, *p*<.001 (see Figure 2A).

The previous analysis prompted further investigation into the interaction effect between word and handedness with the F1-LD-F1 design using multiple comparisons, with the main effect of the handedness on words using two-sample rank-based *t*-test (see Appendix A in File S1).

#### Interaction between word and handedness using multiple comparisons

The interaction effect was analysed sequentially, starting with only the words “up” and “down”, and adding “left” or “right” in the subsequent analysis. Thus it becomes clearer where the interaction effect is initiated. Moreover, given that the interaction is caused by the words in the horizontal direction (“left” and “right”), only the interaction effect with “left” and “right” was additionally analysed. To control the Type I error, the *p*-values after Bonferroni adjustment are reported.

The results are shown in Table 3. They confirmed that the words with horizontal directions cause the interaction, with the word “left” being the most significant followed by “right”, as suggested by the ATS (see Appendix B in File S1).

**Table 3:** Results of the sequential analysis of the interaction between “word” and “handedness” when words were added in the row-wise order reported here.

Interaction “word” and “handedness”	*F* a	Degrees of freedom		*p* b
		Numerator	Denominator	
“up” – “down”	4.40	1	∞	.143
“up” – “down” – “left”	70.26	1.98	∞	<.001
“up” – “down” – “right”	13.38	1.96	∞	<.001
“left” – “right”	137.92	1	∞	<.001

a ANOVA-type statistic.

b *p*-values were adjusted using the Bonferroni correction.

#### Main effect of handedness on each spatial word

As the main effect of handedness appears to be significant, it is of interest to further investigate which spatial word is mainly responsible for the effect. The Brunner-Munzel test for two independent samples [45] and the Munzel test for two dependent samples [46] with their statistics denoted by *W*, were used. In addition, for each test, we report its effect size using the *A* measure of stochastic superiority [47] (see Appendix A in File S1 for details).

#### Ratings of “up” and “down” given by left and right-handers

The Brunner-Munzel test for two independent samples showed that the difference in the ratings for “up” given by left (*M_up-left handers_*  = 2.31, *SE*  = .10) and right-handers (*M_up-right-handers_*  = 2.42, *SE*  = .03) was not statistically significant and showed a very small effect size. The same test showed that the difference in the ratings for “down” given by left (*M_down-left handers_*  = −.88, *SE*  = .14) and right-handers (*M_down-right-handers_*  = −1.15, *SE*  = .04) was not statistically significant and showed a very small effect size. However, the difference in the ratings of “up” and “down” within each handedness group was significant as shown by the non-overlap between 95% confidence intervals (see Figure 2A). Also, using the Munzel test, both the differences in ratings for “up” and “down” for left-handed and for right-handed participants, showed highly statistically significant results with very large effect sizes (see Table 4).

**Table 4:** Pairwise comparisons for the interaction between “word” and “handedness” in Experiment 1.

Pairwise comparison of interest		*W* a	Degrees of freedom	*p*	*A* b
Words	Handedness				
“up”	Left vs Right	1.49	260.39	.14	.54
“down”	Left vs Right	1.79	254.15	.08	.54
“up” vs “down”	Left	23.49	206	<.001	.89
“up” vs “down”	Right	90.46	1945	<.001	.91
“left”	Left vs Right	12.97	244.28	<.001	.74
“right”	Left vs Right	4.38	243.34	<.001	.59
“left” vs “right”	Right	34.65	1945	<.001	.76
“left” vs “right”	Left	2.36	206	.02	.58
*“up” vs “down”*	*All*	*92.81*	*2152*	*<.001*	.*90*
*“left” vs “right”*	*All*	*30.28*	*2152*	*<.001*	.*73*

Comparisons in the “word” factor were carried out using the Brunner-Munzel test for dependent samples and comparisons in the “handedness” factor were carried out using the Brunner-Munzel test for independent samples.

a Brunner-Munzel test for two samples.

b Measure of stochastic superiority (measure of effect size). The interpretation benchmarks are: small∼0.56, medium∼0.64, and large∼.71 (Vargha & Delaney, 2000).

#### Ratings of “left” and “right” given by left and right-handers

The Brunner-Munzel test for two independent samples showed that the difference in the ratings for “left” given by left (*M_left-left handers_*  = 1.52, *SE*  = .14) and right-handers (*M_left-right-handers_*  = −.29, *SE*  = .04) was statistically significant and showed a large effect size (also visually displayed by the non-overlapping 95% confidence intervals). The same test showed that the difference in the ratings for “right” given by left (*M_right-left handers_*  = 1.08, *SE*  = .13) and right-handers (*M_right-right-handers_*  = 1.73, *SE*  = .03) was statistically significant and had a rather small effect size. The difference in the ratings of “left” and “right” in the case of right-handers was statistically significant and showed an extremely large effect size as confirmed by the non-overlap between 95% confidence intervals (see Figure 2A). Since the proportion of non-overlap between confidence intervals for “left” and “right” in the case of left-handers was marginal, the Munzel test was run for further evidence. The test showed that the difference between these ratings was significant but showed a small effect size (see Table 4).

#### Ratings of “up”, “down”, “left”, and “right” across languages and handedness

The Munzel test suggests that the difference in ratings for the words “up” and “down” was significant and showed a large effect size. The same test also showed a significant and large difference in the ratings for the words “left” and “right” (see italicised section in Table 4). The non-overlap between the 95% confidence intervals of the spatial words clearly suggests significantly different ratings between the spatial words (*M_up_*  = 2.42, *SE*  = .03; *M_down_*  = −1.13, *SE*  = .04; *M_left_*  = −.12, *SE*  = .04; and *M_right_*  = 1.67, *SE*  = .03) (see Figure 2B).

### Discussion

The results confirm the hypotheses stated. The effect sizes suggest a salience of the vertical plane over the horizontal plane as seen in each handedness group; however this conclusion would be confirmed in Experiment 2. Interestingly, in this experiment, the effect sizes were quite different between left- and right-handers in the rating of the words “left” and “right”, showing a smaller effect size in the case of left-handers. These results support the idea that left-handers are less lateralised in the horizontal plane than are right-handers. The results also confirmed our predictions in that while left-handers regarded the word “left” as more positive than “right”, this pattern was reversed in the case of right-handers. But, even if left-handers evaluated the word “left” as more positive than “right” (symmetrically to right-handers), they still evaluated “right” as positive, while right-handers evaluated “left” as negative.

The rating task was carried out using a large sample size containing participants from several linguistic backgrounds. The reason several languages were tested was that results were not circumscribed to one language and therefore the ability to generalise based on the findings was assured (see [48]). Thus, the core idea was to provide a general pattern rather than focused analyses about specific-strong hypotheses on the modulation determined by language/culture. Although this experiment aims to provide general patterns rather than focalized analyses of specific-strong hypothesis on the modulation determined by language/culture, at the request of one reviewer, subsamples were compared regarding their writing direction and writing axis in order to find cultural differences. Two post-hoc comparisons were run to investigate this issue. The first comparison involved a pair of cultural groups known for having opposite writing directions on the horizontal axis. An ATS analysis of the between-subjects factors handedness and writing direction of the Italian and Arabic samples showed no significant main effects and interactions of these factors on the ratings of the spatial words (*F _handedness_* (1, 9.56)  = .15, *p = *.70, *F _writing dir_* (1, 9.56)  = 1.52, *p = *.24, and *F _handedness × writing dir_* (1, 9.56)  = .06, *p = *.80). An ATS, with the same factors, of two cultural groups using a ‘leftward’ writing direction (Hebrew and Arabic) and two cultural groups using a ‘rightward’ writing direction (English and German) showed only a significant main effect of this factor (*F _writing dir_* (1, 11.26)  = 12.44, *p = *.004) such that the group ‘Hebrew + Arabic’ gave higher ratings to the words than the group ‘English + German’ (*M _Hebrew + Arabic_*  = 0.52, *SE*  = 0.15, *M _English + German_*  = 0.37, *SE*  = 0.07; *W* = 4.46, *p*<.001, *A* = 0.595). This last result, although significant, does not provide evidence supporting potential cultural differences determined by the writing axis and instead it merely reflects differences in the combined ratings given to spatial words as influenced by handedness (see Figure 1). Should additional significant results emerge with this data, the design of the study itself does not allow a strong conclusion as to cultural differences.

All in all, findings show that individuals who speak different languages (and experience different cultures) behave similarly when judging the valence of direction words. The results suggest a strong linking of space and valenced words, and the dominance of the vertical over the horizontal plane. Additionally, these results further suggest that studies in embodied cognition should not ignore handedness since it is a factor that plays a major role in current embodiment theories.

## Experiment 2

Experiment 1 showed, via a rating task, that the spatial words “up” and “down” were rated as positive and negative, respectively, regardless of handedness, whereas the spatial words “left” and “right” were rated as negative and positive, respectively, only in the case of right-handers. The effect sizes offered a gauge for the difference in the ratings on the vertical and the horizontal planes and suggested a larger discrimination on the vertical plane. Experiment 2 was devised to further these claims by using an implicit word allocation task.

Experiment 2 would thus assist in confirming whether the vertical plane is more salient than the horizontal plane when emotionally-laden words were arbitrarily allocated in space. According to Casasanto [27], different types of interaction with the environment shape the type of mental representations constructed. Consequently, it was expected that a main effect of the horizontal plane would be that, in the case of right-handers, positive words would be placed on the rightward coordinate while negative words would be placed on the leftward coordinate. Such an effect could be coupled with a main effect of the vertical plane as would be expected given the saliency of this plane. However, if the vertical plane had a higher salience than the horizontal plane, it would be expected to find a main effect on the vertical plane only.

### Ethics statement

The experimental protocol was approved by the University of Adelaide and Kyushu University Research Ethics Committees. Following the basic principles of the Declaration of Helsinki, adult participants gave written informed consent.

### Participants

Twenty five English native speakers (16 females, *M_age_*  = 21.28, *SD*  = 5.77, with only 2 left-handers; not included in the analyses), from the University of Adelaide, and 21 Japanese native speakers (8 females, *M_age_*  = 18.6, *SD*  = 3.60, all right-handers), from Kyushu University, took part in the experiment. The criteria adopted to select these two languages were that these samples differ regarding their writing axes (rightward, along the horizontal axis versus downward, along the vertical one).

### Procedure and materials

**Figure 3:**
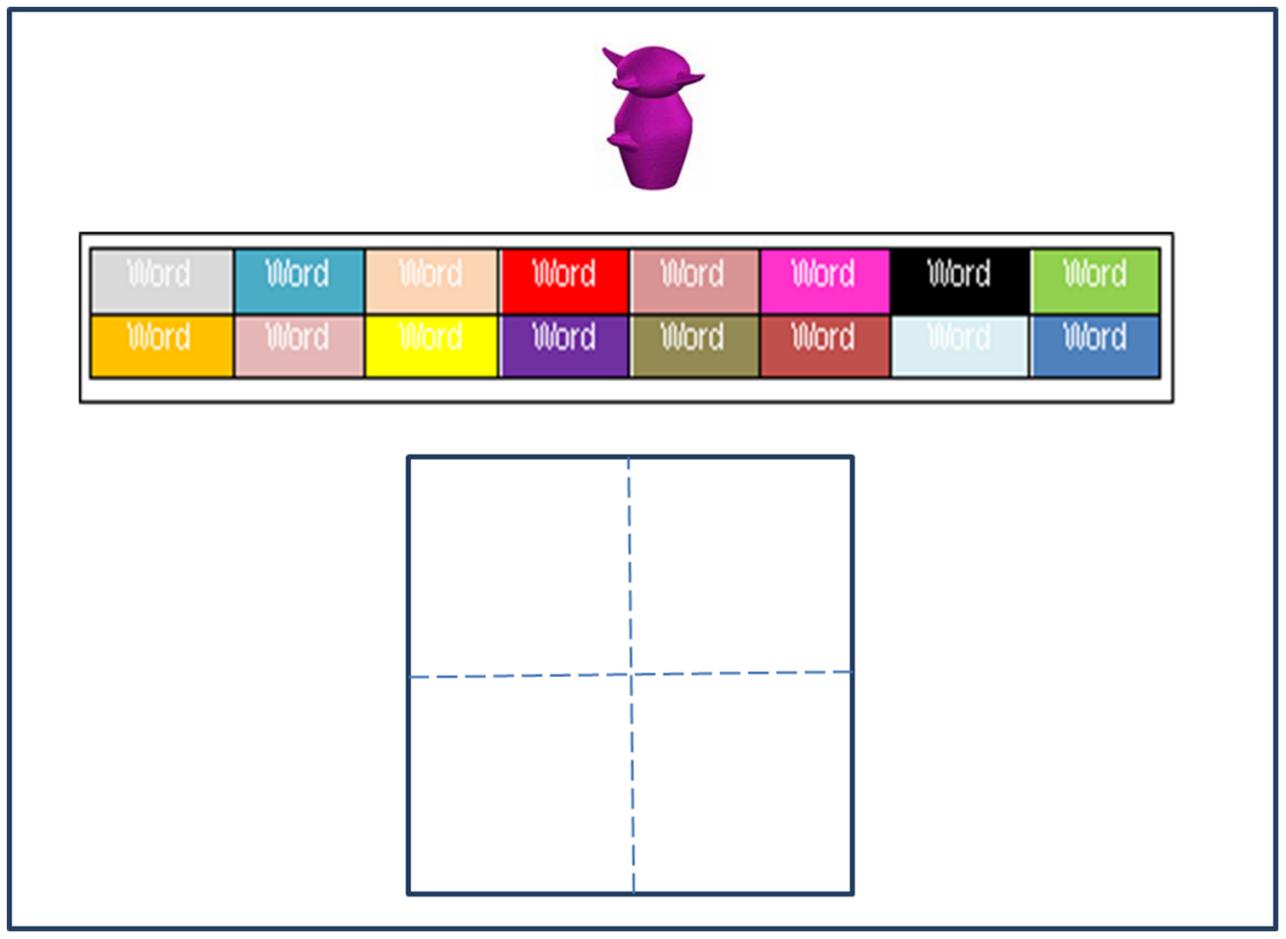
Illustration of the WAT.

Participants were individually seated in front of a computer to perform a novel task labelled here as the “word allocation task” (WAT). The visual distance was approximately 75 cm. In the WAT, participants were presented with a fictional story in which they were invited to assist in the selection of one candidate for a job in a company. To do so, the participants were required to manually allocate personality-trait words via a computer mouse, for each of the candidates, into a squared grid divided into four boxes of equal area (see Appendix C in File S1 listing the 64 personality trait-words selected). Given the wording of the instructions and the fictional setting of the task, the WAT had the advantage of being an implicit task that had very low restrictions. The core instruction given to participants was to arrange the words as they saw fit with the only requirement that they use all of the boxes (explicit instructions and the MATLAB code to run the task can be sent on request). Figure 3 illustrates the display participants viewed during the WAT.

Once participants read the instructions for the task, they were shown the fictional candidates along with their personality-trait words and the allocation grid (11°×11° of visual angle). Participants' tasks consisted of clicking on the word they wanted to allocate in the grid and then clicking on the box in which they wanted to place the word. If the participants changed their mind, they could click on the word just selected and place it in another space. When a word was clicked on, it was highlighted with a red border. Then the participant could click anywhere in the grid and a dot appeared. The colour of that dot was the same colour as the rectangle containing the corresponding word. Each of the 16 words assigned to each fictional character was randomly coloured in each trial. The reasoning behind it was to assist the participant in tracking the word represented by the dot while at the same time avoiding an associating between colour and word over the trials. Although the only condition was that all four boxes in the grid had to be used to allocate the dots representing the words, each box had sufficient space as to accommodate all the dots if required. The four sets of personality trait-words were randomly assigned to each of the fictional characters across participants.

Sixty-four words were taken from a comprehensive list of personality-trait words rated on a 7-point Likert scale (being 0 =  “the least favourable or desirable” and 6 =  “the most favourable and desirable”) regarding their likableness (see [49]). The words selected were categorised into two major groups: low likableness (LL) and high likableness (HL). Within each category, half of the words had high ratings (HR) and the other half had low ratings (LR). Since there were four candidates (fictional characters known as “Greebles”), four sets of 16 personality-trait words were composed. In each set of words, half of the words (8 words) were selected from the LL category and the other half were selected from the HL category. Within each category half of the words (4 words) had HR and the other half had LR (see Table 5). Emotion words and instructions were presented to participants in each linguistic group in Japanese and English.

**Table 5:** Categorisation and mean ratings of the 64 personality-trait words used in the WAT.

Word type	Rating level	Mean rating per rating level (SD)	Mean rating per word type (SD)	95% CI [lower bound, upper bound]
**HL**	**HR**	5.18 (.16)	4.38 (.81)	[4.09, 4.68]
	**LR**	3.59 (.12)		
**LL**	**HR**	2.36 (.13)	1.61 (.76)	[1.33, 1.89]
	**LR**	.86 (.11)		

**Note**. The non-overlap between CIs signals a statistical significant difference between the ratings of the two types of words.

### Design and analysis

The design consisted of one dependent and three independent factors. The mean ratings reported by Anderson [49] for each of the 64 words selected were used as the dependent variable.

The independent factors were vertical location (up and down), horizontal location (left and right), and language (English and Japanese). Vertical and horizontal locations were analysed as within-subject factors and language was analysed as a between-subject factor. Since there were not enough left-handers, handedness was factored out.

In a second analysis, the number of words placed in each location was used as the dependent variable. Only word type was added as a within-subject factor to the model. Given that currently the “nparLD” R package does not have a function to handle 3 within-subjects factors, the SAS MIXED procedure to handle the F1-LD-F3 design was used (see Appendix A in File S1 for the details).

In a final analysis the localised position (X and Y coordinates in visual angles [the actual unit is degrees of arc and is represented by the symbol “°”]) of each word on the computer screen was used as the dependent variable and submitted to a F1-LD-F2 with the factor language, as between-subjects factor or F1, and word type and axes coordinates, as within-subjects factors or F2. Based on the results of this initial analysis, subsequent focused analyses were carried out.

### Results

#### Rating of words and spatial locations

The median rating was computed for each participant in each location combination in order to deal with outlier ratings (see [50]).

The ATS using the F1-LD-F2 design showed only a significant main effect of vertical location, *F* (1, ∞)  = 30.66, *p*<.001 (see Figure 2C). That is, the mean median rating of the words allocated in the “down” location (*M_down_*  = 2.54, *SE*  = .11) was lower than the mean rating of the words allocated in the “up” location (*M_up_*  = 3.35, *SE*  = .10) (*W* = 6.17, *p*<.001, *A* = .70).

The main effects of language, horizontal location, the interaction between vertical and horizontal location, and the interaction between language and locations were not significant (all *p*>.05).

#### Number of negative and positive words and spatial locations

An analysis of the average number of negative and positive words allocated in each of the four possible combinations of vertical and horizontal location, showed a significant interaction between vertical location and word type, *F* (1, ∞)  = 29.04, *p*<.001 (see Figure 2D). This means that more negative words were allocated in the “down” (*M_down-negative_*  = 19.24, *SE*  = .87) than in the “up” location (*M_up-negative_*  = 12.76, *SE*  = .87) (*W*  = −4.20, *p*<.001, *A* = .65), while more positive words were allocated in the “up” (*M_up-positive_*  = 20.98, *SE*  = .83) than in the “down” location (*M_down-positive_*  = 11.02, *SE*  = .83) (*W* = −8.41, *p*<.001, *A* = .75).

Although there was also a main effect of vertical location, *F* (1, ∞)  = 9.10, *p* = .003, in that more words, regardless of their valence and language, were placed in the “up” location (*M_up_*  = 16.87, *SE*  = .74) than in the “down” location (*M_down_*  = 15.13, *SE*  = .74), such difference was not significant (*W* = −1.21, *p* = .23, *A* = .54).

No other main effects or interactions reached significance; all *p*>.05.

#### Words' valence and their localisation on X and Y coordinates

Although the results for the localised positions of the words on the screen showed a marginal main effect of language, *F* (1, 42.7)  = 5.39, *p* = .03, indicating that English speakers placed words, regardless of their valence, in locations whose averaged X and Y coordinates tended to be more positive (*M_English_*  = .49°, *SE*  = .10°) than the location of the words placed by Japanese speakers (*M_Japanese_*  = .24°, *SE*  = .15°), such difference was not statistically significant (*W* = −1.01, *p* = .314, *A* = .56). There was also a main effect of word, *F* (1, ∞)  = 22.15, *p*<.0001, in that negative words were placed in locations whose averaged X and Y coordinates were negative (*M_negative_*  = −.04°, *SE*  = .13°), whereas positive words were placed in locations whose averaged X and Y coordinates were positive (*M_positive_*  = .81°, *SE*  = .09°) (*W* = 5.01, *p*<.001, *A* = .70). A main effect of axes coordinates, *F* (1, ∞)  = 55.38, *p*<.0001, was further substantiated by its interaction with word type, *F* (1, ∞)  = 10.03, *p* = .002. This interaction suggested that negative words were located on negative X coordinates, i.e., leftwards from the centre of the screen, (*M_negative-Xaxis_*  = −.21°, *SE*  = .16°) and Y coordinates close to 0, i.e., towards the centre of the screen (*M_negative-Yaxis_*  = .13°, *SE*  = .21°) (*W* = 1.16, *p* = .25, *A* = .61). Positive words were located on X coordinates close to 0 (*M_positive-Xaxis_*  = −.02°, *SE*  = .13°) and Y coordinates that were above the centre of the screen (*M_positive-Yaxis_*  = 1.62°, *SE*  = .16°) (*W* = 9.60, *p*<.001, *A* = .85) (see Figure 2F). Language interacted significantly with word type, *F* (1, ∞)  = 8.65, *p* = .003, indicating that Japanese speakers allocated negative words in locations whose averaged X and Y coordinates were negative (*M_Japanese-negative_*  = −.44°, *SE*  = .17°), and positive words in locations whose averaged X and Y coordinates were positive (*M_Japanese-positive_*  = .93°, *SE*  = .12°) (*W* = 6.65, *p*<.001, *A* = .86). English speakers allocated negative words in locations whose averaged X and Y coordinates were less positive (*M_English-negative_*  = .30°, *SE*  = .17°) than the averaged X and Y coordinates in which positive words were located (*M_English-positive_*  = .70°, *SE*  = .12°) (*W* = 1.76, *p* = .09, *A* = .56) (see Figure 2E). However, the confidence intervals of positive and negative words in English speakers overlap on the X axis to the point of reaching each other group's mean, thus indicating that the difference may not be significant. Additionally a non-significant three-way interaction between language, word type, and axes coordinates, *p* = .87, also dismisses the idea of a reversed pattern. Finally, the interaction between the language and axes coordinates was not significant, *p* = .08.

### Discussion

The main effect of vertical location confirms the high influence of this axis in the allocation of concepts. This lends support to the differences in effect sizes found between the horizontal and vertical axes reported in Experiment 1. The results obtained in Experiment 2, although based mainly on the performance of right-handers, are in line with the proposal that the vertical plane is more salient than the horizontal plane in relation to the allocation of valenced words, and thus provides evidence in support of our hypothesis. More importantly, positively-valenced words were allocated in the upper areas, while negatively-valenced words were placed in the lower ones; such an allocation strategy did not occur on the horizontal plane.

Participants from two linguistic backgrounds, i.e., English and Japanese, were recruited for this experiment. If there were a significant main effect of the factor “language” on the results, it would have been necessary to run a focused analysis to determine whether linguistic factors could have been the cause. However, the “language” factor turned out to exert no effect on the results. Such a result therefore justifies the generalisation of the present findings to languages other than English and speaks favourably of the robustness of the vertical saliency effect.

It is also notable that English and Japanese native speakers differ for both the ‘writing axes’ (rightward, along the horizontal axis vs. downward, along the vertical one) and potentially for other dimensions affecting emotional experience (see [51]). Therefore, it could be possible that the hypothesis of writing axes might also explain our data but additional data are needed to disentangle this hypothesis.

The results of the X and Y coordinates data suggested some differences between languages. Mainly, the results showed that English speakers placed words, regardless of their valence, in locations whose averaged X and Y values tended to be more positive than the location of the words placed by Japanese speakers. Findings also indicated that English speakers tended to place words in Y coordinates higher than those placed by Japanese speakers. Such results could be attributed to cultural differences or to differences in connotations of words when they were translated. This is certainly an issue that deserves further attention but one that cannot be considered to affect the generalisation of the results presented here. Furthermore, the results shown in Figure 2E might at first suggest a reversed right-positive/left-negative pattern in right-hander English speakers; however, such an idea is dismissed by the overlap of CIs on the X axis and the formal statistical tests. The result of interest is that shown in Figure 2F which lends extra evidence to the findings presented thus far. That is, Figure 2F shows that there is a clear tendency to allocate negative words on leftward locations and positive words on rightward locations, thus consistent with Casasanto [27]. However, there is a larger and significant difference in the allocation of words on the vertical axis in that positive words were placed in locations that lay significantly well above the location of negative words. All in all, these results support the hypothesis presented here and this suggests a saliency of the vertical plane over the horizontal plane in the allocation of emotion words. Further studies using a larger sample are necessary in order to corroborate these results. Additionally, response times could be added as a dependent variable in the WAT used in this study in order to account for the automaticity of the cognitive processes underlying the task.

## General Discussion

The results from the experiments reported above suggest that the vertical plane is more salient than the horizontal plane regarding the allocation of emotion words and positively-valenced words that were placed in upper locations, whereas negatively-valenced words were placed in lower locations. The fact that positive words were allocated in upper locations while negative words were allocated in lower locations is in line with previous studies that have shown associations between vertical positions and positive (for “up”) and negative (for “down”) evaluations (see [22], [25]). However, the finding that the vertical plane is more salient than the horizontal plane in the allocation of concepts is novel. Previous studies have shown that the vertical plane is more salient than the horizontal plane when perceptual and motor tasks are performed (see [33]–[35]). Nevertheless there has been no previous research that has investigated whether the saliency of the vertical plane in the performance of perceptual and motor tasks extrapolates to the conceptual realm. The results of the experiments reported here indicate that this is so.

### Possible mechanisms

This finding thus invites elaboration on the following question: what mechanisms underlie the saliency of the vertical plane over the horizontal plane in the allocation of emotionally-valenced concepts? Given the connection between sensorimotor and metaphorical (affective) systems, three possibilities could be considered as the cause of the vertical salience. The first is a fluent sensorimotor processing on the vertical plane. As reviewed in the introduction, there are many studies (e.g., [33]–[34]) showing the vertical advantage in various types of mental processing, and it is possible that this vertical advantage may occur in the course of adaptation to natural and social environments. In natural environments, the shape of objects, including the human body, and their arrangement in horizontal directions, are symmetric, but they are not always so on the vertical plane. Likewise, Freeman [38] argues that the horizontal axis is less salient as objects in the horizontal axis are easily addressed. This argument is supported by recent findings in attention studies [52]–[55]. Moreover, in social environments the left-right relationship tends to become vague since, as mentioned above, the human body is horizontally symmetric. Thus, information based on the horizontal plane might have a lower informational value than information based on the vertical plane; such low informational value might lead to weaker mappings between sensorimotor systems and emotion metaphors on the horizontal plane than on the vertical plane.

The high informational value of the vertical plane, due to its low uncertainty, might help to increase the saliency of this plane over the horizontal one. For instance, there are cultures in which people write words both from left to right and from right to left (e.g., Arabic, Hebrew, Syriac, or old Japanese). On the other hand, there are cultures in which people write downwards (Chinese, Korean, or Japanese), yet, to the best of our knowledge, cultures using upwards writing direction do not exist. Those instances suggest that the sensorimotor system in the horizontal plane is more ambiguous and plastic than it is in the vertical plane. Indeed, previous research on reversed vision has shown a rapid adaptation to left-right reversed vision compared to upside-down inverted vision, suggesting that the functional plasticity of the sensorimotor system is relatively high in the horizontal plane [56]. Thus, it could be possible that the less-plastic property of vertical sensorimotor processing may develop a strong connection with metaphorical processing.

The second possibility is that linguistic processing mediates the connection between vertical/horizontal spatial metaphors and the sensorimotor system. It has been argued that apraxic patients cannot perform adequate actions with an object even though they can name it and recognise its actions [11]–[12], but it is still unclear whether they can establish a metaphoric mapping of emotion onto space. Moreover, the same issue needs to be further studied using aphasic patients who are unable to name objects. These ideas could be subsumed into the Sapir-Whorf hypothesis that language constrains thinking. Although the Sapir-Whorf hypothesis itself has many loopholes [57], it would be relevant to test the issues of emotional embodiments in apraxic and aphasic patients as the idea of a graded embodiment emphasises to what degree emotions are embodied on a continuum scale, and hence, a correlation with the degree of apraxia and aphasia that could provide valuable information. Such future experimentations will clarify the importance of the strength of the association between abstract concepts on emotion and space. That is, words such as “up” and “down” are coded and used in a consistently positive/negative way in the language and therefore they have a positive/negative association with valence. Instead, words such as “right” and “left” have a less unequivocally valenced coding and use, and therefore they show a reduced effect.

The third is the involvement of an attention-based mechanism. It is possible that the observer's attention shifted to locations that are congruent with a metaphorical mapping between word and space [58]. The attention shift induces relatively high evaluation to items in the upper space, compared to items in the lower space, due to attentional devaluation [59]–[61]. Furthermore, attention attracts localization of an item toward attended locations [62]–[64]. The biased localization of emotionally-laden items that we demonstrated in Experiment 2 could have been a product of this attentional attraction. Thus, an attention-based explanation would seem to be reflected in our results as well as in previously found evidence relating to the relationship between emotion and space. Importantly, this explanation has a hidden assumption that attention shift congruent with a metaphorical mapping dominantly occurs in the vertical plane rather than the horizontal one. Unfortunately, little evidence on this issue has been provided. Therefore, further examination for this is needed to clarify the role of attention in connecting emotion and space.

### Theoretical implications for embodiment processing

The results reported here have implications for research in spatial cognition, emotion, and psycholinguistics. These areas are further connected when the viewpoint of embodied cognition theory is added. In the case of spatial cognition research, recent evidence showing a saliency of the vertical plane over the horizontal plane in the performance of perceptual and motor tasks was reviewed. Studies in haptic, visual, and auditory processing provide evidence for this claim. However, evidence from the latter should be interpreted cautiously. As reviewed above, while some evidence suggests a salience of the vertical auditory plane, under very specific experimental situations [35], other evidence suggests a saliency of the horizontal plane [36]. Given the physical organisation of the auditory system, it could be predicted that in particular sensory systems, there should be a higher salience of the horizontal plane. To the best of our knowledge, a saliency of the vertical plane over the horizontal plane has not been determined in the gustatory and olfactory sensory modalities. The reason that there is no evidence for this is probably because testing the saliency of one plane over the other might seem simply irrelevant and/or difficult to test.

The results are also congruent with evidence from emotion research that shows associations between positive concepts and upward and rightward space locations, while negative concepts are associated with downward and leftward locations. The association on the vertical plane is expected to occur in the case of both left- and right-handers, whereas the association on the horizontal plane is expected to occur only in the case of right-handers. In the case of left-handers, an opposite pattern in the horizontal plane is expected, i.e., positive concepts are associated with leftward locations. Differences in the association between emotion concepts and the horizontal plane, as determined by handedness, are predicted based on the findings of Casasanto [27], whereas the association between emotion concepts and the vertical plane, independent from handedness, seems to be a generic prediction. Most of the studies that show these patterns are carried out using off-line tasks, as is the case with those reported here. Therefore it cannot be affirmed that these associations are automatic on both planes (although see [65]) for evidence based on a Stroop task which supports an automatic association between emotion concepts and the vertical plane). Novel uses of priming tasks have shown an automatic activation of sensorimotor representations to spatial words (see [66]). Hence, a potential research avenue would be to adapt these new priming tasks to determine whether the association between emotion concepts and spatial locations is automatic and under what conditions the association might occur (for instance, it has been shown that such associations do not always occur automatically [67]).

More importantly, what is still open to question is whether the person's current emotional state might alter such associations, i.e., most studies assume participants are in a rather neutral emotional state. However, it has not been specifically determined whether, for example, a right-hander in a sad emotional state at the time of the experiment would indifferently allocate negative concepts on both leftward and rightward space locations. Indeed, studies along these lines would assist in corroborating claims from vision research, which suggests that whereas reversed adaptation in the horizontal plane is malleable, adaptation in the vertical plane is not (see [56]). In hypothetical visual adaptation tasks aimed at addressing this question, participants' emotional states could be elicited prior to their response to emotionally-valenced images presented on the vertical and horizontal planes. In the case of haptic and auditory tasks, emotionally-valenced manipulable objects and emotionally-valenced sounds could be linked to vertical and horizontal locations in space.

Finally, the results reported here have implications for psycholinguistic research, particularly in relation to cross-linguistic studies of emotion and spatial cognition. The purpose of Experiment 1 was to identify the general trend across several languages in the rating of spatial works. In Experiment 1 analyses based on specific languages were not selected since the original intention of the rating task was not to compare the performances between linguistic groups. The main finding of Experiment 1 is that, across various languages, the prediction based on the findings of Casasanto [27] holds, and there is also evidence to suggest a saliency of the vertical plane over the horizontal plane. That is, the effect sizes signal a stronger discrimination in the appraisal of spatial words referring to the vertical plane than to spatial words referring to the horizontal plane. The results of Experiment 2 confirmed this trend, across two languages, thus providing evidence in support of our hypothesis. In summary, the results obtained in both experiments substantiate the generalisation of the present results to speakers of languages other than English (see [48]).

On a methodological and cautionary note, it should be noted that most tasks use laboratory experimental tasks which may have a reduced relation to cognitive process that unfold in everyday situations. Although various recent experimental tasks have proposed novel and intellectually sophisticated methodologies for the study of embodied cognition theories, the findings may be valid exclusively within this experimental situation. Therefore, it is relevant to engineer tasks that allow for situated cognitions (see [68]) which are ecologically valid. This problem can be addressed by devising on-line and off-line tasks that require complete body movements in relation to the experimental materials (e.g., emotionally-valenced items). These sorts of tasks already exist and have been used to study how body postures relate to memory recall (e.g., [69]), to emotionally-valenced images (e.g., [70]), and to problem solving tasks (e.g., [71]), among others. However, a task of this kind has not yet been devised to account for the association between emotion concepts and spatial locations and for the study of the saliency of one spatial plane over the other. The results from these studies could provide evidence in relation to more context-dependent cognitive processes.

## Conclusions

The results presented provide supporting evidence to the BSH hypothesis consistent with a previous study [27]. The prediction based on BSH was that while right-handers regarded the word “right” as more positive than “left”, left-handers regarded the word “left” as more positive than “right”. However, they also provide complementary and novel evidence suggesting a salience of the vertical plane over the horizontal plane at the concept level (the word “up” was evaluated more positively than “down”). That is, the evidence reviewed suggests a saliency of the vertical plane over the horizontal plane in the performance of perceptual and motor tasks, although it has not yet been investigated whether this saliency would occur in the processing of concepts. The present study shows that such a saliency advantage occurs at the conceptual level, specifically in the case of emotionally-valenced concepts. The results of these studies extend prior recent work (e.g. [67]) suggesting that an association between physical space and emotional valence requires a task with an explicit response mapping to occur. Although several ideas were presented as to why this might occur, more research is needed in order to substantiate the current claims. Furthermore, it is vital that tasks devised to answer questions in relation to the topics addressed thus far are tested across various languages and are ecologically valid in order to determine the everyday-life relevance of the findings.

## Supporting Information

Figure S1 **The RTE plot showing the probability that a randomly chosen observation in the subset of the data tends to be larger than a randomly chosen observation in the whole data.**
(TIF)

Table S1 **The personality-trait words extracted from** **Table 1** **in Anderson (1968).**
(PDF)

File S1 **Supporting Appendices.**
(DOCX)
