# Supplementary figures and images for: Appraisal of Space Words and Allocation of Emotion Words in Bodily Space
Source: PLoS One. 2013 Dec 11;8(12):e81688. doi: 10.1371/journal.pone.0081688 (PMC3859505; doi:10.1371/journal.pone.0081688)

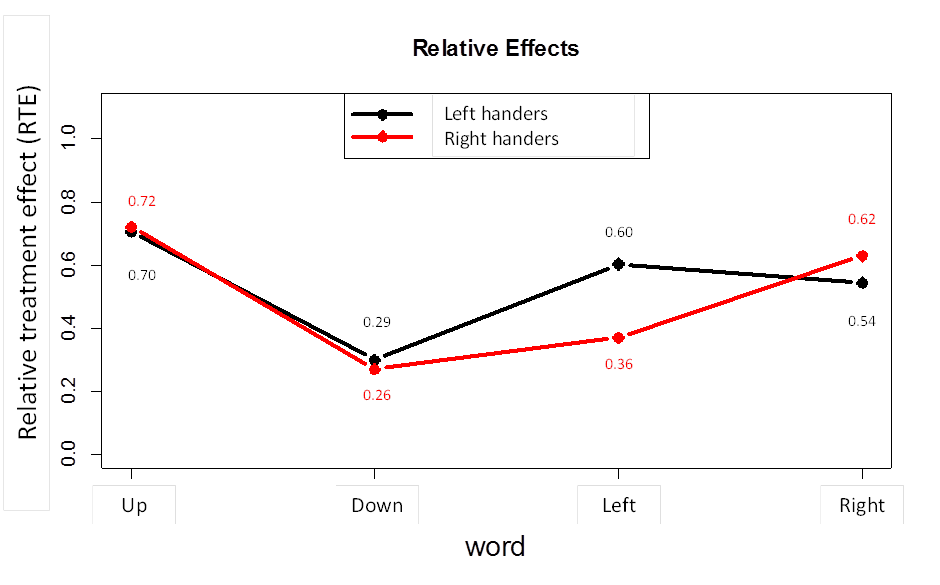

Supplement: Figure S1 — The RTE plot showing the probability that a randomly chosen observation in the subset of the data tends to be larger than a randomly chosen observation in the whole data. (TIF) [file pone.0081688.s001.tif]
