# Supplementary material for: Appraisal of Space Words and Allocation of Emotion Words in Bodily Space
Source: PLoS One. 2013 Dec 11;8(12):e81688. doi: 10.1371/journal.pone.0081688 (PMC3859505; doi:10.1371/journal.pone.0081688)
Supplement: Table S1 — The personality-trait words extracted from Table 1 in Anderson (1968). (PDF) [file pone.0081688.s002.pdf]

Word type	Sub-classification	Rating	Word
**HR**	**HR-HR$_s$**	5.45	Truthful
		5.39	Trustworthy
		5.37	Intelligent
		5.36	Dependable
		5.3	Open-minded
		5.29	Thoughtful
		5.28	Wise
		5.27	Considerate
		5.04	Cheerful
		5.04	Trustful
		5.04	Warm-hearted
		5.03	Broad-minded
		5.03	Gentle
		5.01	Well-spoken
		5	Educated
		5	Reasonable
	**HR-LR$_s$**	3.74	Persuasive
		3.73	Obedient
		3.73	Quick
		3.72	Sophisticated
		3.72	Thrifty
		3.71	Sentimental
		3.7	Objective
		3.69	Nonconforming
		3.51	Satirical
		3.48	Prudent

		3.48	Reserved
		3.47	Persistent
		3.46	Meticulous
		3.46	Unconventional
		3.45	Deliberate
		3.45	Painstaking
**LR**	**LR-HR$_s$**	2.54	Dependent
		2.53	Unsystematic
		2.49	Self-conscious
		2.49	Undecided
		2.48	Resigned
		2.47	Clownish
		2.46	Anxious
		2.46	Conforming
		2.25	Uninquisitive
		2.24	Forgetful
		2.24	Inhibited
		2.24	Unskilled
		2.23	Crafty
		2.23	Passive
		2.22	Immodest
		2.22	Unpopular
	**LR-LR$_s$**	1	Abusive
		0.99	Distrustful
		0.98	Intolerant
		0.98	Unforgiving
		0.97	Boring

0.97	Unethical
0.97	Unreasonable
0.96	Self-centred
0.79	Vulgar
0.78	Heartless
0.78	Insolent
0.77	Thoughtless
0.76	Rude
0.74	Conceited
0.72	Greedy
0.72	Spiteful
