# Supplementary material for: Appraisal of Space Words and Allocation of Emotion Words in Bodily Space
Source: PLoS One. 2013 Dec 11;8(12):e81688. doi: 10.1371/journal.pone.0081688 (PMC3859505; doi:10.1371/journal.pone.0081688)
Supplement: File S1 — Supporting Appendices. (DOCX) [file pone.0081688.s003.docx]

**Appendix A**

Traditionally, ordinal data, including Likert-type data, obtained via factorial designs, have been analysed using repeated measures ANOVA. This statistical model assumes that the data are continuous and that the covariance matrix has a specific structure called sphericity which includes the compound symmetry structure as a special case [72, p. 266]. In the case of Likert-type data, continuity is clearly violated and an appropriate structure of the covariance matrix is often unknown prior to the experiment [73]. Therefore, the results obtained may not be reliable, for example, when the data clearly show non-homogeneous variances across different levels of a dependent factor.

To overcome such a problem, it has become popular to analyse data in the framework of linear mixed models (LMM) (e.g., [74]) with various covariance matrix structures. Repeated measures ANOVA are considered a special case of LMM [75, p. 345], which assumes sphericity of the covariance matrix. However, LMM allows for various covariance matrix structures, including unstructured covariance matrix, which is a reasonable choice if the data structure is unknown a priori. Yet, fitting large data to a complex linear mixed model is often faced with nonconverging iteration processes, making the results unreliable (see [76, p. 50]). In Experiment 1 there was an effect of non-converging iteration problems by comparing the results obtained from SAS and R when the present data was fitted to LMM (with 8612 observations). For SAS, the PROC MIXED was used, and for R, the “lmer” function from the “lme4” package was used.

However, recent work in statistics has proposed a robust rank-based nonparametric alternative that deals with mixed designs for a broad class of data, including Likert-type and continuous data (e.g., reaction times), without a specific assumption of the covariance matrix [77-80]. Such an alternative, called the ANOVA-type statistic (or ATS) for factorial designs, also provides reliable results for small sample sizes and data with outliers [81]. The ATS is used to test the hypothesis of the equality of marginal distributions, rather than the equality of means (see [78, p. 40]) and has wide applications to data arising from psychological experiments such as reaction time data for probe words (see Example 1 in [82]). Moreover, the nonparametric models accommodate various higher-way layouts of the F*x*-LD-F*y* type, where *x* and *y* represent the number of between-subjects and within-subjects factors, respectively (see Chapter 10 in [78]). Although the textbook by Brunner et al. [78] mainly focuses on longitudinal data to illustrate the use of ATS, mathematically, there is no distinction between repeated measures data and longitudinal data in factorial designs without any assumption on the covariance matrix structure. In fact, in such designs, longitudinal data are considered to be in the class of repeated measures data with “time” as a within-subjects factor [83]. Therefore, the use of ATS in the experiments presented here is valid and, in fact, provides more reliable conclusions compared to the classical repeated measures ANOVA (even with the Greenhouse-Geisser or the Huynh-Feldt correction; see [83] for more discussion).

Thus, in Experiment 1, ratings were analysed using a nonparametric mixed 4 × 2 ANOVA in the framework of an F1-LD-F1 design. The inferences about the significance of these factors were made based on the ATS for the F1-LD-F1 design, currently implemented in the R statistical software “nparLD” and SAS-IML macro library. The function “f1.ld.f1” in the “nparLD” R package was used to perform the analyses. The function was entered in this way:

| f1.ld.f1(rating, word, handedness, ID, time.name = “word”, group.name = “handedness”). |
| --- |

In Experiment 2, the design known as a F1-LD-F2 was used [78]. In this design, F1 denotes the only between subjects factor (language), F2 denotes two within-subjects factors (vertical and horizontal location) and LD represents the dependent variable. The function “f1.ld.f2” in the “nparLD” R package was used to perform the analyses. The function was entered in the following way:

| f1.ld.f2(median_rating, vert, horiz, lang, sub, time1.name=“vert”, time2.name=“horiz”, group.name =“lang”, description=TRUE, time1.order=NULL, time2.order=NULL, plot.RTE=TRUE, show.covariance=TRUE). |
| --- |

For the rank-based nonparametric ANOVA, the data are firstly transformed to (mid-) ranks, and statistics are calculated based on these ranks. When the data are transformed to ranks, the assumption of homoscedasticity does not hold in general [84], and hence the conventional degrees of freedom, which are derived from the assumption of homoscedasticity, may not produce accurate results. To accommodate for such a problem, Box-type approximations [81] are applied to approximate the distribution of the ATS. The Box-type approximations can be seen as an extension of the well-known Satterthwaite-Smith-Welch degrees of freedom adjustment for two-sample *t*-test with unequal variances, and are equivalent to the conventional degrees of freedom calculations for equal variances (homoscedastic) cases [81]. For the within-subjects factor (e.g., the factor “word” in Experiment 1) and interactions involving the within-subjects factor, approximations using the numerator degrees of freedom are available while the denominator degrees of freedom is assumed to be infinity. This is due to the fact that a finite denominator degrees of freedom tends to provide very conservative results with an increase in the number of levels in the within-subjects factor, leading to a low power [83]. For the between-subjects factors and their interactions, more accurate results can be obtained by using both the numerator and denominator degrees of freedom approximations. Hence, throughout the paper, the approximated degrees of freedom are given instead of the conventional degrees of freedom obtained from the traditional repeated measures ANOVA.

Following the idea above, a similar analysis may be carried out for the F1-LD-F3 design as was done in Experiment 2. However, since this design is not available in the “nparLD” R package, SAS MIXED procedure may be used instead to calculate the statistics and the corresponding *p*-values. Prior to calculation, it is necessary to transform the data into ranks. Once the ranked data are obtained, the function can be entered in the following way.

| proc mixed data=ranked.data method=mivque0 anovaf;  CLASS lang word VL HL sub;  MODEL response = lang\| word\| VL\| HL/CHISQ;  Repeated word*VL*HL / SUB=sub TYPE=UN GRP=lang;  LSMEANS lang*word*VL*HL;  run; |
| --- |

Using similar formats, it is theoretically possible to carry out analysis for higher layouts, i.e., in the case where *x* and *y* are greater than 2 in the F*x*-LD-F*y* design.

After significant effects are identified from the nonparametric ANOVA, multiple comparisons for the interaction effects and pair-wise comparisons for the main effects may be performed. For example, for Experiment 1, finding the interaction effects also rely on the ATS with the F2-LD-F1 design, with some levels omitted in order to identify the level responsible for such interactions.

For the pair-wise comparisons of main effects, the Brunner-Munzel test for two independent samples and the Munzel test for stochastic equality ([46], [78 p. 93, equation (7.6)]) for two dependent samples were applied. Although traditionally Wilcoxon rank sum tests or Wilcoxon signed rank tests are applied as nonparametric two-sample tests, they are not recommended due to their strict assumptions and non-robustness to unequal variances [45, 85]. The Brunner-Munzel test is implemented in the R package “lawstat”, and the function was entered in the following way:

| brunner.munzel.test(x, y, alternative = "two.sided", alpha = 0.05) |
| --- |

Where the vectors *x* and *y* are the data from the two samples. For the Munzel test, the code was implemented as follows:

| two.sample.bf<-function(x,y)  {  n<-length(x)  rx<-rank(x)  ry<-rank(y)  r<-rank(c(x,y))  rxc<-r[1:n]  ryc<-r[(n+1):(2*n)]  rxm<-mean(rxc)  rym<-mean(ryc)  s2np<-(1/(n-1))*sum(((ryc-ry)-(rxc-rx)-(rym-rxm))^2)  Tpn<-sqrt(n)*(rym-rxm)/(2*sqrt(s2np))  p.value<-2*pt(abs(Tpn),df=(n-1),lower.tail=FALSE)  df<-n-1  return(list(statistic=Tpn, p.value=p.value, df=df))  } |
| --- |

And the function was entered in the following way:

| two.sample.bf(x,y) |
| --- |

Additionally, an EDA approach for pair-wise comparisons (see [86]) was used. In particular, the focus was on what overlapping and non-overlapping 95% confidence intervals indicated (see [87-88]). Confidence intervals were adjusted for the case of within-subjects measures using the Loftus and Masson [89] method (see also [90]).

Finally, an estimator of effect size (ES) called measure of stochastic superiority (denoted as *A*, see [47]) was used in the analysis. The *A* is a nonparametric ES for dependent and independent multiple-wise comparisons that can be used on discrete and continuous variables, thus it is appropriate for the current analyses. Also, the idea of stochastic superiority is consistent with the Munzel test for stochastic equality. In the two samples case, *A* measures the difference between two populations in terms of the probability that a score randomly taken from population *1* will be greater (or smaller) than a score randomly taken from population *2*. The magnitude of the difference can be interpreted on a scale ranging from 0.5 to 1, with .56 being a small effect size, .64 a medium effect size, and .71 a large effect size. Note that *A* can be shown to be mathematically equivalent to Cliff’s [91] delta (see Equation 9 in [47]). An extension to multiple-wise comparisons using *A* is called “*relative treatment effects (RTEs) (or relative effects)*” [78, p. 38] in which each sample is compared to the average of all marginal distributions.

We implemented two versions of *A*, namely, the two independent sample case and matched correlated sample case. The two samples are denoted by *x* and *y*. We provide the code below.

Two independent sample case:

| a12<-function(x,y)  {  m<-length(x)  n<-length(y)  x.greater<-function(xelem,y)  {  xglen<-length(which(xelem>y))  return(xglen)  }  x.equal<-function(xelem,y)  {  xelen<-length(which(xelem==y))  return(xelen)  }  xgvec<-tapply(x,c(1:m),x.greater,y=y)  xevec<-tapply(x,c(1:m),x.equal,y=y)  a<-sum(xgvec)/(n*m)+0.5*sum(xevec)/(n*m)  superiority<-"x is superior to y."  if(a < 0.5)  {  a<-1-a  superiority<-"y is superior to x."  }  return(list(a=a,superiority=superiority))  } |
| --- |

Matched correlated sample case (see (51) in [47]):

| a51<-function(x,y)  {  m<-length(x)  n<-length(y)  if(m!=n)  {  stop("x and y must have the same length.")  }  diff<-x-y  num.equal<-sum(as.numeric(diff==0))  num.greater<-sum(as.numeric(diff>0))  a<-(num.greater+0.5*num.equal)/n  superiority<-"x is superior to y."  if(a < 0.5)  {  a<-1-a  superiority<-"y is superior to x."  }  return(list(a=a,superiority=superiority))  } |
| --- |

**Appendix B. RTE (relative treatment effect) plot of the interaction between word and handedness (Experiment 1).**

Figure S1 shows the probability that a randomly chosen observation in the subset of the data (e.g., rating of “up” by left handers) tends to be larger than a randomly chosen observation in the whole data (see [42, p. 7]). For example, the RTE of 0.70 for “up” given by left handers means that a randomly chosen rating for “up” by left handers would be larger than a randomly chosen rating in the whole dataset with a probability of approximately 70%.

There is a noticeable difference in RTEs between left and right handers for the word “left” (0.60 and 0.36, respectively). These numbers can be understood as follows: the left handers tend to give a much higher rating for the word “left” than the right handers. The opposite can be concluded for the word “right”. Also, there is not much difference in RTEs for “up” and “down” between left and right handers, suggesting that the ratings for these two words are similar between these two groups. As a remark, the RTE plot may be thought of as an analogous measure to the mean in parametric ANOVA.

**Appendix C. The sixty four personality trait-words used in Experiment 2**

Table S1 shows the personality-trait words extracted from Table 1 in Anderson [49]. Ratings were performed on a 7-point Likert scale ranging from 0 (“the least favourable or desirable”) to 6 (“the most favourable or desirable”). Anderson presents a histogram (see Figure 1 in his paper) that suggests that ratings tended to cluster around the range of 1 to 2 and 4 to 5, while there was a dip around the value of 3, i.e. at the centre of the scale. Thus, the overall distribution of the ratings was a bimodal distribution in that there were words with low and high ratings. The high-rating words (here HR) were those located in the range of 3 to 6, while low-rating words (here LR) were those located in the range of 0 to 3. A sub-classification was made within each distribution of HR and LR words in such a way that low-rating and high-rating words were included in the list of experimental words. Thus, high-rating words in the HR distribution (here HR-HRs) were those close to the upper end of the distribution, while low-rating words in this distribution (here HR-LRs) were those words close to the lower end of the distribution. Likewise, high-rating words in the LR distribution (here LR-HRs) were those close to the upper end of the distribution, while low-rating words in this distribution (here LR-LRs) were those words close to the lower end of the distribution.

**References**

72. Bakeman, R., & Robinson, B. F. (2005). Understanding statistics in the behavioral sciences. Mahwah, NJ: Erlbaum.

73. Li, J., Xia, Y., Palta, M., & Shankar, A. (2009). Impact of unknown covariance structures in semiparametric models for longitudinal data: An application to Wisconsin diabetes data. Computational Statistics and Data Analysis, 53(12), 4186-4197

74. Baayen, R.H., Davidson, D.J., & Bates, D.M. (2008). Mixed-effects modeling with crossed random effects for subjects and items. Journal of Memory and Language, 59, 390-412.

75. Dixon, W. J. (1985). BMDP statistical software: 1985 Printing. University of California Press.

76. Verbeke, G., & Molenberghs, G. (2000). Linear Mixed Models for Longitudinal Data. Springer.

77. Brunner, E., Munzel, U., & Puri, M.L. (1999). Rank-score tests in factorial designs with repeated measures. Journal of Multivariate Analysis, 70, 286-317.

78. Brunner, E., Domhof, S., & Langer, F. (2002). Nonparametric analysis of longitudinal data in factorial experiments. N. Y.: Wiley.

79. Shah, D. A., & Madden, L. V. (2004). Nonparametric analysis of ordinal data in designed factorial experiments. Phytopathology, 94 (1), 33-43.

80. Kapstein, M., Nass, C., & Markopoulos, P. (2010). Powerful and consistent analysis of Likert-type rating scales. CHI 2010 Proceedings of the 28th international conference on Human factors in computing systems (pp. 2391-2394). Atlanta, Georgia: Association for Computing Machinery.

81. Brunner, E., Dette, H., & A. Munk (1997). Box-type approximations in nonparametric factorial designs. Journal of the American Statistical Association, 92 (440), 1494-1502.

82. Akritas, M. G., & Brunner, E. (1997). A unified approach to rank tests in mixed models. Journal of Statistical Planning and Inference, 61, 249–277.

83. Bathke, A.C., Schabenberger, O., Tobias, R.D., & Madden, L.V. (2009). Greenhouse-Geisser adjustment and the ANOVA-type statistic: Cousins or twins? The American Statistician, 63, 239-246.

84. Akritas, M. G. (1990). The rank transform method in some two-factor designs. Journal of the American Statistical Association, 85 (409), 73-78.

85. Neuhauser, M., & Ruxton, G. D. (2009). Distribution-free two-sample comparisons in the case of heterogeneous variances. Behavioral Ecology and Sociobiology, 63 (4), 617-623.

86. Yu, C. H. (2010). Exploratory data analysis in the context of data mining and resampling. International Journal of Psychological Research, 3(1), 9-22.

87. Cumming, G. (2009). Inference by eye: Reading the overlap of independent confidence intervals. Statistics in Medicine, 28, 205-220.

88. Marmolejo-Ramos, F., & Matsunaga, M. (2009). Getting the most from your curves. Exploring and reporting data using informative graphical techniques. Tutorials in Quantitative Methods for Psychology, 5 (2), 40-50.

89. Loftus, G. R., & Masson, M. E. J. (1994). Using confidence intervals in within-subject designs. Psychonomic Bulletin & Review, 1, 476–490.

90. Marmolejo-Ramos, F., & Tian, S. (2009). The shifting boxplot. A boxplot based on essential summary statistics around the mean. International Journal of Psychological Research, 3 (1), 37-46.

91. Cliff, N. (1993). Dominance statistics: Ordinal analyses to answer ordinal questions. Psychological Bulletin, 114, 494-509.
